# The Crossroads of the Coagulation System and the Immune System: Interactions and Connections

**DOI:** 10.3390/ijms241612563

**Published:** 2023-08-08

**Authors:** Grzegorz Wilhelm, Paulina Mertowska, Sebastian Mertowski, Anna Przysucha, Jerzy Strużyna, Ewelina Grywalska, Kamil Torres

**Affiliations:** 1 Department of Plastic and Reconstructive Surgery and Microsurgery, Medical University of Lublin, 20-059 Lublin, Poland; grzegorz.wilhelm@op.pl (G.W.); kamil.torres@umlub.pl (K.T.); 2 Department of Experimental Immunology, Medical University of Lublin, 20-093 Lublin, Poland; sebastian.mertowski@umlub.pl (S.M.); ewelina.grywalska@umlub.pl (E.G.); 3 Chair and Department of Didactics and Medical Simulation, Medical University of Lublin, 20-093 Lublin, Poland; annaprzysucha15@gmail.com; 4 East Center of Burns Treatment and Reconstructive Surgery, Medical University of Lublin, 20-059 Lublin, Poland; jerzy.struzyna@umlub.pl

**Keywords:** immune system, coagulation system, sepsis, autoimmune diseases

## Abstract

The coagulation and immune systems, two vital systems in the human body, share intimate connections that fundamentally determine patient health. These systems work together through several common regulatory pathways, including the Tissue Factor (TF) Pathway. Immune cells expressing TF and producing pro-inflammatory cytokines can influence coagulation, while coagulation factors and processes reciprocally impact immune responses by activating immune cells and controlling their functions. These shared pathways contribute to maintaining health and are also involved in various pathological conditions. Dysregulated coagulation, triggered by infection, inflammation, or tissue damage, can result in conditions such as disseminated intravascular coagulation (DIC). Concurrently, immune dysregulation may lead to coagulation disorders and thrombotic complications. This review elucidates these intricate interactions, emphasizing their roles in the pathogenesis of autoimmune diseases and cancer. Understanding the complex interplay between these systems is critical for disease management and the development of effective treatments. By exploring these common regulatory mechanisms, we can uncover innovative therapeutic strategies targeting these intricate disorders. Thus, this paper presents a comprehensive overview of the mutual interaction between the coagulation and immune systems, highlighting its significance in health maintenance and disease pathology.

## 1. Introduction

The immune system recognizes and eliminates pathogens, promotes inflammation, and coordinates immune responses [1,2,3,4]. It consists of innate and acquired immunity. The innate immune system provides the first line of defense by responding rapidly to pathogens through pattern recognition receptors (PRRs), including Toll-like receptors (TLRs), which detect pathogen-associated molecular patterns (PAMPs). This recognition triggers immune responses including cytokine and chemokine production, immune cell recruitment, and complement activation [3,4,5,6]. On the other hand, the coagulation system is primarily responsible for maintaining hemostasis, preventing excessive bleeding, and promoting wound healing. It involves a cascade of reactions leading to the formation of a blood clot, primarily through the activation of coagulation factors and platelets [7,8,9]. The coagulation system is tightly regulated to ensure clot formation when needed and is appropriately controlled to avoid thrombosis [10,11,12]. The interconnectedness of the coagulation system and immune systems’ interconnectedness is evident in pathological conditions [11]. Excessive or dysregulated activation of coagulation can occur in response to infection, inflammation, or tissue damage, leading to disseminated intravascular coagulation (DIC). Dysregulated immune responses, as seen in conditions such as sepsis, may promote coagulopathy and microvascular thrombosis [13,14]. Moreover, this coagulation–immunity relationship is implicated in various diseases beyond hemostasis and inflammation. Emerging evidence suggests that dysregulated coagulation may contribute to the pathogenesis of autoimmune diseases, cancer, and chronic inflammatory disorders. Conversely, immune dysregulation may promote a prothrombotic state, increasing the risk of venous and arterial thrombosis [15,16,17,18].

Therefore, this review aimed to present the interactions between the coagulation system and the immune system in maintaining health and its involvement in the pathogenesis of immune disorders and autoimmune diseases.

## 2. Coagulation System—Mechanism of Action

The coagulation system, also known as the blood coagulation system, is a complex biological process that regulates hemostasis, which is the body’s ability to control bleeding. It is a complex enzyme system that responds to damage to blood vessels by forming blood clots at the site of injury [7,19,20]. Hemostasis is a complex physiological process that involves four stages to stop bleeding and maintain blood within the vascular compartment following vascular injury. The first stage is vascular constriction, which is induced immediately after vascular injury to limit blood flow and reduce hemorrhage. This is facilitated by neurogenic mechanisms and endothelin release. The second stage is platelet adhesion and activation, which is instigated by the exposure of the sub-endothelial matrix to circulating platelets. The von Willebrand factor and integrins like GPIb facilitate the adhesion of platelets to the damaged vessel wall. The engagement of collagen and thrombin with platelet surface receptors initiates platelet activation and aggregation, leading to the formation of a platelet plug. The third stage is the coagulation cascade, which consists of the intrinsic, extrinsic, and common pathways. This stage is critical to the process because it activates the cascade of proteolytic activations of coagulation factors [7,19,20]. Thrombin is generated during this stage, which is integral to the coagulation phase. The prothrombinase complex, comprising activated Factor X and Factor V in the presence of calcium ions on a phospholipid surface, converts prothrombin to its active form. Thrombin subsequently catalyzes the transformation of soluble fibrinogen to insoluble fibrin monomers, which polymerize to form a fibrin meshwork that stabilizes the platelet plug. The fourth and final stage is clot retraction and vessel repair. Post-fibrin mesh formation, platelets within the clot contract, is facilitated by the actin–myosin complex, leading to clot retraction [7,19,20]. This draws the edges of the damaged vessel together to support tissue repair. Concurrently, the process of fibrinolysis is initiated to carefully balance clot formation and resolution. In summary, thrombin generation is integral to the coagulation phase, not only catalyzing fibrinogen to fibrin conversion but also promoting platelet activation and aggregation. However, the coagulation phase also encompasses the activation of the coagulation cascade and the subsequent steps of fibrin polymerization and clot stabilization [19,20].

**Figure 1:**
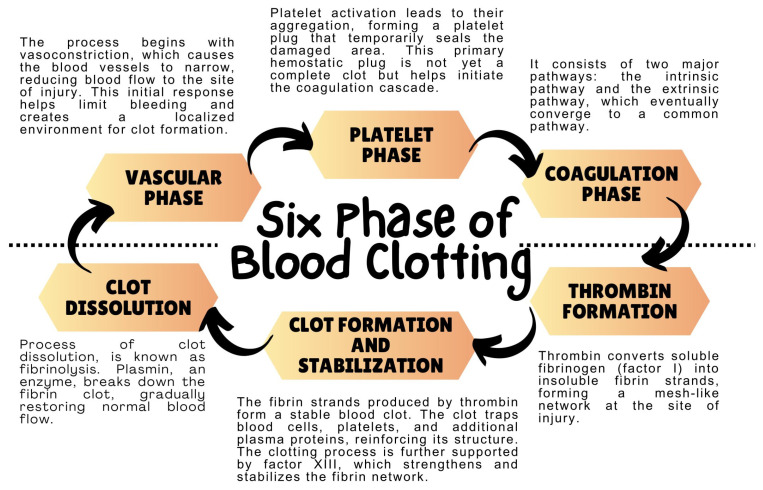
Clot formation and dissolution phases based on [8].

The coagulation system can be divided into several main components. The first is platelets, which are tiny, irregularly-shaped cell fragments found in the bloodstream. They play a crucial role in blood clotting. When a blood vessel is damaged, platelets become activated and form clots that help seal the injury and prevent excessive bleeding. Derived from large precursor cells called megakaryocytes in the bone marrow, platelets are disc-shaped without a nucleus. They contain granules and organelles inherited from the megakaryocytes. Platelets release various substances to promote clotting and facilitate wound healing. They are essential for maintaining hemostasis and aiding in the repair of injured blood vessels. Another component present in blood is blood plasma, which contains numerous coagulation factors responsible for initiating and regulating the coagulation process. These factors consist of fibrinogen, prothrombin, coagulation factors (such as factor VIII, factor IX, and factor X), and anticoagulation factors (such as protein C and protein S). Hemostasis, also known as the blood clotting process, involves a series of intricate steps aimed at controlling bleeding and facilitating the formation of a stable blood clot. The process of blood coagulation encompasses various phases, including the vascular phase, platelet phase, coagulation phase, thrombin formation, clot formation, stabilization, and subsequent dissolution (Figure 1) [8,19].

The coagulation phase is particularly critical and can be further subdivided into several pathways [21]. The first pathway is the internal pathway, which is activated when a blood vessel is damaged, leading to the exposure of collagen beneath the endothelium. This process requires the involvement of multiple coagulation factors, such as factor XII, factor XI, and factor IX [22,23]. The second pathway is the extrinsic pathway, which is triggered by external tissue injuries that release tissue factor (TF) when there is tissue injury or damage, and immune cells such as monocytes and macrophages recognize the presence of foreign substances or damaged cells. Then, the TF serves as a critical initiator of the coagulation process. TF interacts with factor VII, a clotting factor present in the blood, and forms a complex known as the TF/factor VIIa complex. This process is essential for preventing excessive bleeding and promoting wound healing. TF binds to factor VII, activating it and forming a complex with TF VIIa [23]. These two pathways converge into a common pathway involving the conversion of factor X to factor Xa by factor V and calcium ions. Factor Xa then converts prothrombin (factor II) to thrombin (factor IIa). Thrombin plays a crucial role in the coagulation process as it converts fibrinogen (factor I) into fibrin [23].

The coagulation system operates as a tightly regulated process involving numerous coagulation factors and regulatory molecules. The intricate interplay between the intrinsic and extrinsic pathways, combined with the final steps of thrombin formation and fibrin clot generation, guarantees effective hemostasis and the maintenance of blood flow, while simultaneously averting excessive bleeding [24,25]. It is crucial to acknowledge that any disruption to this process may result in coagulation disorders or thrombotic complications. Thus, a comprehensive understanding of the course of coagulation is paramount for the successful management and treatment of bleeding disorders.

## 3. Blood Coagulation Process and the Role of Immune System Cells

Although the primary role of the immune system is to recognize and defend against pathogens, it also plays a significant role in the regulation of coagulation. Immune system cells are essential for forming the initial platelet plug and play an important role in initiating and regulating the coagulation process [26,27,28]. Particular attention in handling coagulation mechanisms deserves not only platelets but also other immune system cells, such as monocytes, neutrophils, lymphocytes, or dendritic cells, whose detailed characteristics are presented in the following subsections. In addition, as research shows, the coagulation system actively interacts with the immune system, modulating immune responses. Coagulation factors such as thrombin can activate immune cells and increase the production of pro-inflammatory cytokines. Fibrin, the main component of blood clots, acts as a scaffold for immune cells and promotes their recruitment and activation at the site of injury or infection [27,29,30,31].

### 3.1. Platelets

Platelets are not only essential for the formation of the initial platelet plug but also play a crucial role in activating the coagulation cascade [32]. When platelets come into contact with exposed collagen at the site of injury, they become activated and release substances that recruit and activate other immune system cells. The main substances found in platelet granules are dense granules, α granules, and cytosol substances.

Dense granules contain small molecules involved in platelet aggregation, vasoconstriction, and the recruitment of additional platelets to the injury site. The substances found in dense granules include adenosine diphosphate (ADP); adenosine triphosphate (ATP); serotonin (5-hydroxytryptamine); and calcium ions (Ca^2+^) [33,34]. The next group are alpha granules. They are larger and more numerous than dense granules. They contain various proteins involved in platelet adhesion, blood clotting, inflammation, and tissue repair. The substances found in alpha granules include Von Willebrand factor (vWF); fibrinogen; coagulation factors (such as Factor V, Factor VIII); platelet-derived growth factor (PDGF); transforming growth factor-beta (TGF-β); platelet factor 4 (PF4); fibronectin; thrombospondin; and β-thromboglobulin [35,36,37]. In the case of cytokines and chemokines occurring within the platelets, we can distinguish such compounds as interleukin-1 β, CD40 ligand, CCL2, CCL3, CCL5, CXCL1, CXCL4, CXCL12, andCXCL16 [38,39]. Platelet cytosol contains various coagulation factors, ions, and other signaling molecules involved in platelet activation, shape change, and platelet plug formation. Essential substances present in the platelet cytosol include actin and myosin (contractile proteins); glycolytic enzymes (for energy production); phospholipases (e.g., phospholipase A2); protein kinases (involved in intracellular signaling); calcium ions (Ca^2+^); cyclic adenosine monophosphate (cAMP); nitric oxide (NO); and reactive oxygen species (ROS) [40,41,42]. Platelets also provide a surface for collecting clotting factors and facilitate clot formation. The interaction between blood coagulation and platelets is a dynamic process that involves a delicate balance between procoagulant and anticoagulant factors to maintain hemostasis. One such molecule is protease activated receptor (PAR), which is activated after proteolytic cleavage by various coagulation factors, including thrombin and other coagulant proteases. PAR activation leads to the release of pro-inflammatory mediators such as cytokines, chemokines, and adhesion molecules, thus amplifying the inflammatory response. In addition, PARs can also induce the production of TF, a critical initiator of the coagulation cascade. By initiating a two-way signaling pathway between coagulation and inflammation, PARs serve as a key link between the two systems [43,44,45,46]. PARs also interact with Toll-like receptors (TLRs), a type of PRR, to regulate inflammation. TLRs are critical in the early innate immune response to invading pathogens, recognizing the pathogen and initiating inflammatory responses. Upon activation, PARs can either upregulate or downregulate TLR expression, thereby modulating TLR-mediated pathogen recognition and subsequent inflammatory responses [47,48].

PARs also regulate the downstream signaling pathways of TLRs, including the activation of nuclear factor-kappa B (NF-kB) and the production of type I interferons, both of which are crucial to inflammatory and antiviral responses [48]. For example, in bacterial infections, bacterial proteases can activate PARs, leading to the production of proinflammatory cytokines and chemokines that recruit immune cells to the site of infection. Similarly, in viral infections, activation of PARs can modulate TLR3 and TLR7/8 signaling, which is essential for the recognition of viral RNAs and subsequent antiviral immune response [49]. The other most important platelet receptors, which are crucial for maintaining normal hemostatic function, are presented in Table 1 [43,44,45,46].

**Figure 2:**
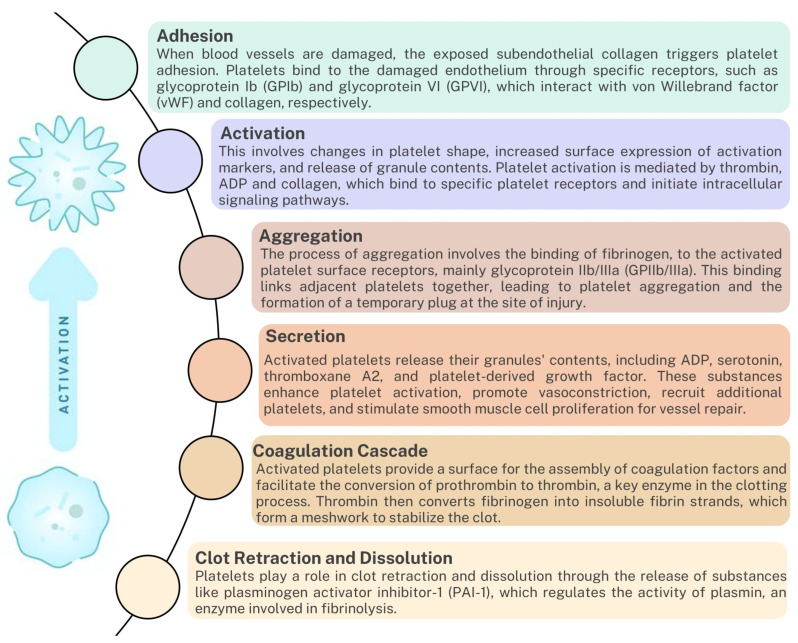
The role of platelets in the coagulation cascade and clot formation based on [64,65].

Also, platelets play a crucial role in regulating coagulation by releasing various bioactive molecules (Figure 2). One such molecule is polyphosphates, which are negatively charged polymers consisting of multiple phosphate units. Polyphosphates are abundant in platelet-dense granules and significantly enhance the activation of the intrinsic pathway of coagulation [63]. They activate factor XII (FXII) and initiate the contact pathway by promoting FXII autoactivation. Once activated, FXII activates downstream coagulation factors, such as factor XI (FXI) and factor IX (FIX), thus amplifying the coagulation response. The presence of polyphosphates in platelets provides an additional layer of regulation to the coagulation system by promoting intrinsic pathway activation and modulating hemostatic processes. In summary, dysregulation of platelet function can lead to blood coagulation disorders or hypercoagulation, highlighting the importance of this intricate interaction in maintaining vascular health [42,63,64,65,66].

### 3.2. Tissue Factor (TF)

Tissue Factor (TF) is a transmembrane protein that is crucial for initiating the coagulation cascade process which forms a blood clot to stop bleeding after an injury. TF, also known as thromboplastin, coagulation factor III, F3, or (CD142), is a transmembrane glycoprotein receptor found on various cells. It has a molecular weight of 47 kDa and functions as a receptor for coagulation factors VIIa and X. When bound to FVIIa, TF can initiate blood coagulation [67,68]. TF acts as a receptor for coagulation Factor VII (FVII) and can bind to it when FVII circulates in its inactive form (FVIIa). This binding triggers a series of events that activate other clotting factors and lead to the formation of a fibrin clot. The location of TF expression determines its function [67,68]. Under normal conditions, TF is expressed on the surface of cells lining the vessel walls, acting as a protective mechanism to control bleeding upon injury. This form of TF, known as “hemostatic TF,” initiates the clotting process only when the vessel wall integrity is compromised. Conversely, under pathological conditions, TF is expressed by circulating cells such as monocytes and microparticles, contributing to excessive clot formation and leading to thrombosis. It is worth mentioning that extracellular vesicles such as microparticles or exosomes carrying TF or phosphatidylserine (PS) are significant players in coagulation [69,70]. These vesicles, often released by cells during activation or apoptosis, carry TF or PS on their surface. PS becomes externalized on these vesicles, providing a catalytic surface for the assembly of coagulation complexes [69,70]. This phenomenon can potentially amplify the clotting process, particularly in pathological situations such as cancer, sepsis, and autoimmune diseases. Understanding the different roles and functions of TF is crucial in developing targeted therapies for various bleeding and clotting disorders [67,68,70].

### 3.3. Monocytes/Macrophages

**Figure 3:**
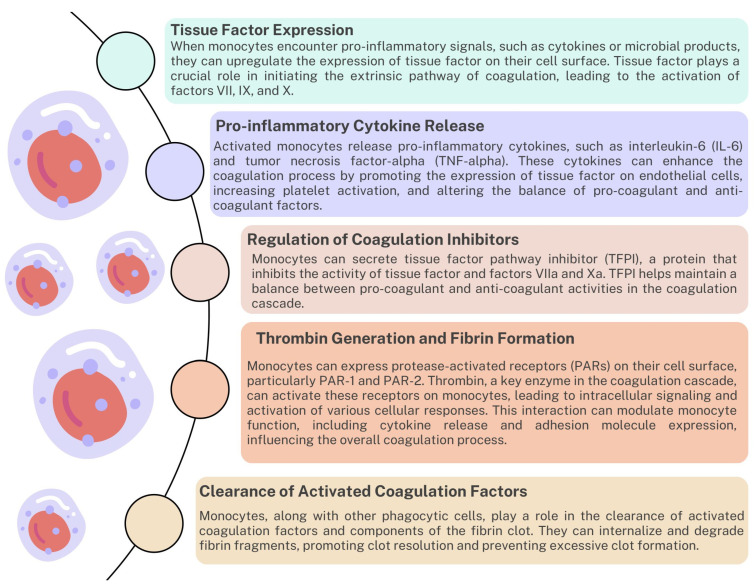
The role of monocytes in the coagulation system based on [72,73,74].

**Figure 4:**
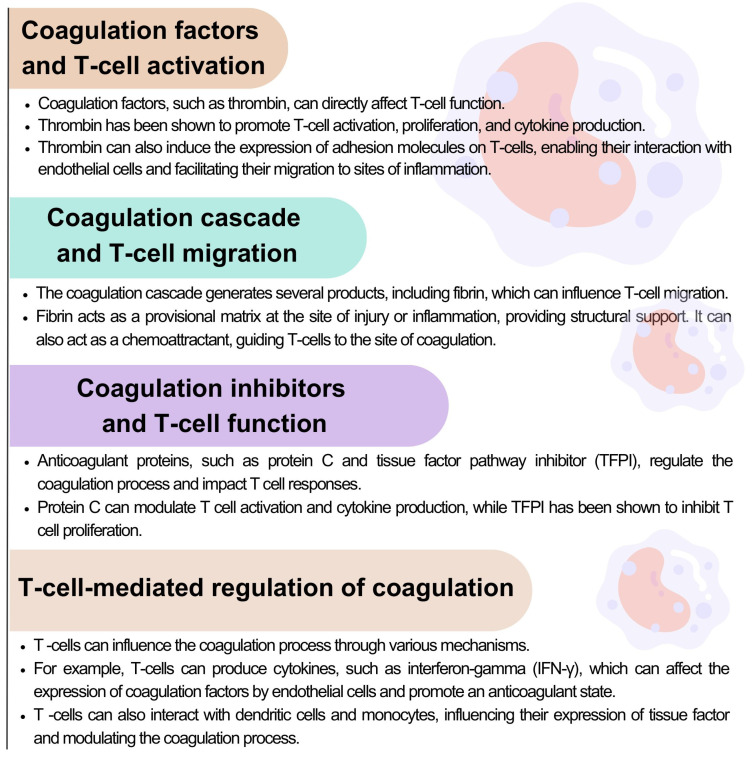
Interactions between the coagulation system and T lymphocyte activation, migration, and function based on [132,133,134].

Monocytes, a type of white blood cell, differentiate into tissue macrophages. Macrophages release pro-inflammatory cytokines and chemokines that contribute to initiating and regulating the coagulation process (Figure 3) [71]. They also express TFs necessary to activate the extrinsic coagulation pathway. They are involved in regulating coagulation inhibitors, thrombin generation, fibrin formation, and clearance of activated coagulation factors (Figure 4) [72,73,74]. Additionally, monocytes contribute to the complicated interaction between coagulation and inflammation. When tissue damage or infection occurs, monocytes are recruited to the site of inflammation. They migrate from the bloodstream to inflamed tissues in response to chemotactic signals released by damaged cells or activated endothelium. This initial recruitment of monocytes is essential in initiating the inflammatory response. At the site of inflammation, monocytes become activated and release pro-inflammatory cytokines such as tumor necrosis factor-alpha (TNF-alpha), interleukin-1 (IL-1), and interleukin-6 (IL-6). These cytokines play a crucial role in promoting a local inflammatory response. They increase the recruitment of other immune cells, increase vascular permeability, and stimulate the production of acute-phase proteins by the liver [75,76]. 

Activated monocytes interact with the endothelial cells lining the blood vessels at the site of inflammation. They bind to the endothelium through adhesion molecules such as selectins and integrins. This interaction promotes the transmigration of monocytes across the endothelial layer, allowing them to enter inflamed tissues [77,78,79]. Then, in the tissues, monocytes differentiate into macrophages, which have an increased capacity for phagocytosis. Macrophages can engulf and remove cellular debris, pathogens, and other foreign particles present at the site of inflammation [80,81]. They also function as antigen-presenting cells, presenting processed antigens to activate other immune cells such as T cells. Monocyte-derived macrophages secrete many pro-inflammatory mediators, including cytokines, chemokines, and growth factors. These molecules recruit and activate other immune cells, such as neutrophils and lymphocytes, to enhance the inflammatory response [82]. The best-known cytokines secreted by monocytes that are involved in the coagulation process include: IL-1 (can upregulate the expression of TF on endothelial cells, monocytes, and other cell types, leading to an increased procoagulant state), IL-6 (can stimulate the production of fibrinogen, a key component of blood clots, by the liver, thus promoting coagulation), IL-8 (attracts and activates neutrophils which can release factors that promote coagulation), and TNF-α (can enhance platelet activation and aggregation) [29,75,76,77,78,79,80,81,82,83]. This amplification is important for effectively eliminating pathogens and promoting tissue repair. As the inflammatory response progresses, monocytes/macrophages play a role in the resolution phase of inflammation. They produce anti-inflammatory cytokines such as interleukin-10 (IL-10) and TGF-β, which help suppress the immune response and promote tissue healing [76,77,78,79,80,81,82,83,84,85,86,87,88]. Monocytes/macrophages also participate in removing apoptotic cells and tissue debris, contributing to the resolution of inflammation. Due to the diverse functions that monocytes or macrophages perform during the development of inflammation and regulation of the coagulation cascade and tissue regeneration, dysregulation of their functions may affect coagulation disorders and thrombotic diseases such as Deep Vein Thrombosis (DVT) [74,89,90] and Pulmonary Embolism (PE) [90,91], Arterial Thrombosis [92,93], Disseminated Intravascular Coagulation (DIC) [94], Antiphospholipid Syndrome (APS) [95,96], or Thrombotic Microangiopathies [97,98], highlighting the importance of understanding their interaction with the coagulation system (Table 2).

### 3.4. Neutrophils

Neutrophils are another type of white blood cell that plays a role in blood clotting. They are recruited to the site of injury or inflammation and release neutrophil extracellular traps (NETs) [100,101,102,103]. These NETs comprise DNA, histones, and antimicrobial proteins that trap pathogens and promote clot formation. NETs contain various procoagulant factors such as tissue, histones, and platelet-activating factors. These ingredients can promote the activation of the coagulation cascade and contribute to the formation of blood clots. However, excessive formation of NETs may also contribute to the development of thrombotic disorders [104,105,106,107]. Moreover, like other cells of the immune system, neutrophils can interact with platelets to form heterotypic aggregates mediated by adhesion molecules and receptors on both neutrophils and platelets, such as P-selectin and integrins [108,109,110,111,112]. These aggregates can promote platelet activation and aggregation, increasing blood clots’ formation. In addition, they also participate in the modulation of endothelial function due to the release of pro-inflammatory cytokines and chemokines, which may disrupt the integrity of the endothelial barrier and promote the activation of endothelial cells, which, as a consequence of increased expression of adhesion molecules, may lead to the development of thrombosis—another mechanism in which neutrophils are involved in removing activated coagulation factors and components of the fibrin clot. Studies also indicate that neutrophils contribute to the resolution of inflammation and tissue repair processes, which are necessary for proper wound healing [101,113,114,115,116]. The interaction between neutrophils and the blood coagulation system is complex and highly regulated. Although neutrophils promote coagulation, leasing procoagulant factors and forming NETs, they also contribute to clot dissolution and tissue repair. Dysregulation of these interactions can result in pathological conditions such as excessive coagulation or impaired wound healing.

### 3.5. Dendritic Cells

The interaction between blood coagulation and dendritic cells (DCs) is a complex and multifaceted relationship [117,118,119]. DCs are a type of immune cell that play a key role in both innate and adaptive immune responses. They are known for their ability to capture antigens and present them to other immune cells, thus initiating immune responses [120,121]. DCs also contribute to regulating the coagulation process, which is accomplished by releasing pro-inflammatory cytokines and promoting platelet activation [112]. Additionally, DCs express several coagulation factors on their surface, such as TF and thrombomodulin, which regulate the blood coagulation cascade by binding thrombin and activating protein C, an anticoagulant [122,123]. Moreover, studies have shown that thrombin can induce maturation and activation of DCs, leading to increased antigen presentation and recruitment of immune cells. Another compound involved in DC activation is fibrin, which can affect DC antigen presentation and modulate T cell activation and polarization, which is essential in regulating the immune response [124,125].

### 3.6. Lymphocytes

Lymphocytes are white blood cells that play a key role in the immune system. There are two main types of lymphocytes: T lymphocytes (T cells) and B lymphocytes (B cells), which differ in their functions in the human body [126]. T lymphocytes are primarily responsible for cellular immune responses. They mature in the thymus and are divided into several subsets based on the presence of specific cell surface proteins known as cluster differentiation (CD) markers. Significant subsets of T cells include Helper T cells (CD4+ T cells), Cytotoxic T cells (CD8+ T cells), and Regulatory T cells (Tregs). The first subpopulation recognizes antigens presented by antigen-presenting cells (APCs) and releases a cytokine to activate other immune cells, such as B cells and cytotoxic T cells. Helper T cells are crucial for generating antibody responses (humoral immunity) and activating cellular immune responses. The second subpopulation involves cellular immune responses against infected or tumor cells. They recognize and kill target cells by releasing cytotoxic molecules such as perforin and granzymes. Cytotoxic T cells are essential in eliminating intracellular pathogens and tumor cells. The third subpopulation helps maintain immune homeostasis and prevents excessive immune reactions. These cells suppress immune reactions and regulate the activity of other immune cells, preventing autoimmunity and overactivation of the immune system [127,128]. The second type of lymphocyte is B cells, primarily responsible for humoral immune responses. They mature in the bone marrow and differentiate into plasma cells that produce antibodies. B cells can recognize antigens directly or by interacting with helper T cells. After antigen recognition, B cells undergo clonal expansion, differentiation into plasma cells, and antibody production. Antibodies secreted by plasma cells bind to specific antigens, marking them for destruction or neutralizing their activity. B cells also have antigen-presenting abilities because they can internalize and present antigens to helper T cells [129,130]. In addition to such essential and diverse functions in the human body’s immune responses, research has shown that lymphocytes, including B cells and T cells, interact with platelets and affect the coagulation process. B cells can produce antibodies that recognize and bind to platelet antigens, leading to platelet activation and aggregation. T lymphocytes can modulate the immune response and the production of pro-inflammatory cytokines, affecting the balance of procoagulant and anticoagulant factors [131].

#### 3.6.1. The Role of T Lymphocytes in the Coagulation Process

The interplay between blood coagulation and T lymphocytes highlights the intricate relationship between the immune and hemostatic systems. Coagulation factors can influence the function and migration of T cells. In contrast, T cells can mutually influence the coagulation process by producing cytokines and interacting with other cells involved in hemostasis (Figure 4) [132,133,134].

Understanding these interactions is critical to unraveling the complex mechanisms underlying immune responses and thrombotic disorders. Studies have shown that T lymphocytes are involved in the development and progression of thrombotic events in certain pathological conditions, such as deep vein thrombosis and atherosclerosis [135,136,137]. During thrombus formation in DVT, the endothelial cells lining the blood vessels are damaged, releasing inflammatory molecules and recruiting immune cells, including T cells. These T cells are attracted to the site of thrombosis by chemokines and adhesion molecules [136]. Once recruited to the site of thrombosis, T cells can become activated and produce various cytokines. Studies have shown that activated T cells, in particular CD4+ T cells, release pro-inflammatory cytokines such as interferon-gamma (IFN-γ) and TNF-α, which further promote inflammation and contribute to the development of DVT [137,138,139]. Research suggests that specific subsets of T cells, such as Tregs, may play a protective role by modulating the inflammatory response, promoting tissue repair, and facilitating thrombus clearance [140,141,142]. T lymphocytes may influence the vascular remodeling processes that occur during the recovery phase of DVT. It may promote the formation of new blood vessels (neovascularization) and contribute to vessel wall remodeling, which may have long-term implications for venous function and the risk of recurrent thrombosis [143,144,145]. Scientific research shows that T lymphocytes can interact with endothelial cells, platelets, and other immune cells, creating a prothrombotic environment [146,147]. Although the exact mechanisms underlying the involvement of T cells in DVT are still being investigated, understanding the role of these cells in the development and progression of DVT may lead to the development of new therapeutic strategies for this disease.

#### 3.6.2. The role of B Lymphocytes in the Coagulation Process

**Figure 5:**
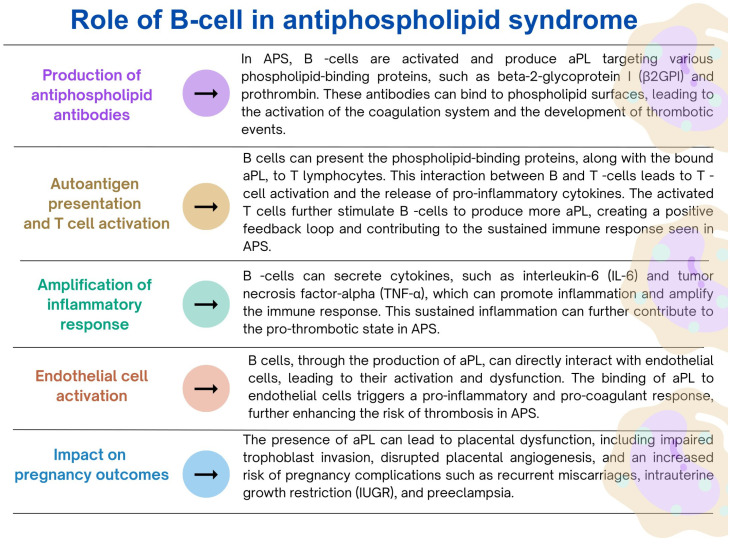
The role of B lymphocytes in the development and progression of the antiphospholipid syndrome based on [155,156,157].

**Figure 6:**
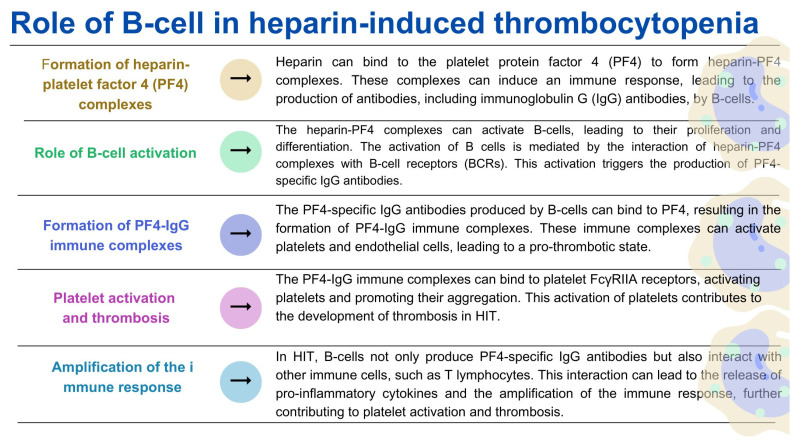
The role of B lymphocytes in heparin-induced thrombocytopenia based on [158,159,160,161].

B lymphocytes are immune cells that play a key role in adaptive immunity, especially in the production of antibodies [148]. Moreover, as indicated by recent research, these cells are also involved in regulating the blood coagulation process. B lymphocytes are primarily responsible for producing antibodies, including antibodies specific to coagulation factors [149]. These antibodies can regulate the activity of coagulation factors, such as antiphospholipid antibodies, which interfere with phospholipids’ function in the coagulation cascade [150]. Dysregulation of this process can lead to autoimmune conditions associated with abnormal coagulation and antibody production. In addition, B lymphocytes, mainly a subset called B-1 cells, are involved in dissolving blood clots. These B cells can produce natural antibodies that bind to the fibrin component of the lump and facilitate its degradation [30]. Studies have also shown that coagulation factors such as thrombin and fibrinogen can directly affect B cell activation. Thrombin has been shown to stimulate B cell proliferation and antibody production. In addition to its role in clot formation, fibrinogen can interact with B lymphocytes via integrins, affecting B lymphocyte adhesion and migration [30,151,152,153,154]. So, what happens if B lymphocytes’ normal functioning in the coagulation system context is dysregulated? As indicated in the literature, deregulation of the immune response mediated by B lymphocytes can lead to the development of thrombotic disorders, and more specifically to antiphospholipid syndrome (APS) (Figure 5) [155,156,157] and heparin-induced thrombocytopenia (HIT) (Figure 6) [158,159,160,161]. 

Understanding the role of B cells in APS or HIT is important in order to discover the mechanisms underlying these immune-mediated disorders. The researchers indicate that further research targeting B cell activation and the production of pathogenic antibodies may, in the future, result in a new personalized therapeutic approach for the treatment of APS. Moreover, the literature data show that targeting B-cell activation and producing pathogenic antibodies has been explored as a potential therapeutic strategy in treating HIT [162,163]. Researchers also indicate that further identification of ways to modulate B cell responses may help prevent or treat HIT-related thrombotic complications.

It is important to note that although immune system cells play an essential role in the blood clotting process, dysregulation or overactivation of these cells can contribute to pathological coagulation, such as autoimmune diseases or sepsis. Crosstalk between the coagulation and immune systems is complex and tightly regulated to maintain a delicate balance between clot formation and prevention of excessive clotting.

## 4. The Role of the Coagulation System in Selected Disorders of the Immune System

### 4.1. Role of Immunotrombosis

Immunothrombosis is a multifaceted process that involves the crosstalk between the immune system and the coagulation system. It represents an emerging concept in thrombosis research, highlighting the active participation of immune cells, soluble mediators, and cellular interactions in the formation and regulation of thrombi [164].

Immunothrombosis encompasses the intricate interplay between the immune system’s innate and adaptive components and the coagulation cascade. Immune cells have been implicated in immunothrombosis, including neutrophils, monocytes, macrophages, and platelets. These cells express a wide array of receptors, such as Toll-like receptors (TLRs), C-type lectin receptors (CLRs), and Fc receptors, enabling them to recognize danger signals, microbial pathogens, and immune complexes. Upon activation, immune cells release pro-inflammatory cytokines, chemokines, and other soluble mediators, creating a microenvironment that promotes thrombus formation [164,165,166].

Neutrophils, in particular, play a crucial role in immunothrombosis. They can release extracellular traps known as neutrophil extracellular traps (NETs), which are composed of chromatin fibers decorated with antimicrobial peptides, proteases, and histones. NETs can ensnare pathogens and form a scaffold for platelet adhesion and activation, thereby promoting thrombus formation. Moreover, neutrophils can directly interact with platelets and endothelial cells, further amplifying the immune-driven thrombotic response [101,167,168]. Monocytes and macrophages are also integral players in immunothrombosis. These immune cells can recognize pathogens or damaged tissue through pattern recognition receptors (PRRs) and phagocytose them. In doing so, monocytes and macrophages release TF, a key initiator of the coagulation cascade, leading to local activation of the clotting system and thrombus formation. Additionally, macrophages secrete various cytokines and growth factors that modulate endothelial function and contribute to the prothrombotic milieu [92].

Platelets, traditionally recognized for their role in hemostasis, also actively participate in immunothrombosis. They express an array of immune receptors, including TLRs and Fc receptors, enabling them to sense and respond to immune stimuli. Upon activation, platelets release chemokines, cytokines, and von Willebrand factor (vWF), which facilitate immune cell recruitment and promote their interactions with the vessel wall, ultimately contributing to thrombus formation [169,170]. In addition to immune cells, the complement system plays a critical role in immunothrombosis. The complement system consists of a cascade of enzymatic reactions that can be activated via classical, lectin, or alternative pathways. Activation of the complement system leads to the generation of anaphylatoxins, opsonins, and the formation of the membrane attack complex (MAC), all of which can trigger pro-inflammatory and prothrombotic responses. In summary, Immunothrombosis represents a tightly regulated process that is crucial for effective host defense and tissue repair. However, dysregulation of immunothrombotic responses can contribute to the pathogenesis of various thrombotic disorders.

### 4.2. Interrelations between Complement and Hemostasis

The complement system and hemostasis are complex biological systems that play vital roles in immune responses and coagulation. The interplay between complement and hemostasis involves intricate molecular interactions and cross-regulation, which contribute to the maintenance of immune homeostasis and hemostatic balance [171].

Complement activation can influence various aspects of the coagulation cascade. For example, components of the complement system, including C3a and C5a, can directly stimulate platelet activation, leading to enhanced platelet adhesion and aggregation. Furthermore, complement activation fragments can promote pro-inflammatory and procoagulant responses, affecting the production and activity of coagulation factors. The anaphylatoxins generated during complement activation can modulate endothelial cells, platelets, and leukocytes, thereby influencing the hemostatic process [171,172,173].

Conversely, components of the coagulation system can also impact complement activation and regulation. For instance, TF, a key initiator of the coagulation cascade, can trigger the complement pathway, leading to the generation of anaphylatoxins and the formation of the membrane attack complex (MAC) [174]. Moreover, activated platelets and endothelial cells release complement-regulatory proteins that modulate the activity of complement components, exerting control over immune and hemostatic processes [172,173,174]. Disruption or dysregulation of either the complement or coagulation pathways can have significant implications for thromboinflammatory conditions. Excessive complement activation can create a prothrombotic environment through the generation of anaphylatoxins, the assembly of the MAC, and interference with fibrinolysis. This dysregulated complement activation can contribute to the pathogenesis of disorders such as thrombotic microangiopathies, disseminated intravascular coagulation (DIC), and venous thromboembolism [171,174]. Similarly, dysregulation of the coagulation system can directly impact complement activation and exacerbate inflammation. Coagulation factors, particularly thrombin, can activate complement components, triggering an inflammatory cascade. Imbalances in the coagulation system, including endothelial dysfunction and platelet activation, can enhance complement activation and subsequent thromboinflammatory responses [171,174]. Understanding the interrelations between complement and hemostasis has important clinical implications for the management of thromboinflammatory disorders. Targeting specific components or pathways involved in complement activation or coagulation holds promise for therapeutic interventions aimed at restoring the delicate balance between these systems.

### 4.3. Coagulation Disorders and Sepsis

**Figure 7:**
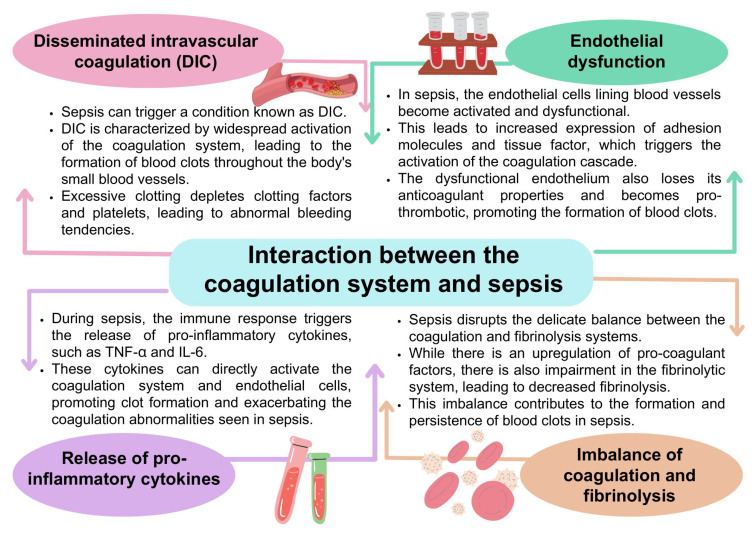
Coagulation disorders in the course of sepsis based on [13,178,179,180,181,182].

The coagulation system and sepsis are closely related [175,176,177]. Sepsis is a life-threatening condition caused by a dysregulated immune response to infection. The interaction between the coagulation system and sepsis is complex. It involves many elements, such as DIC, endothelial dysfunction, coagulation, fibrinolysis imbalances, and the release of pro-inflammatory cytokines (Figure 7) [13,178,179,180,181].

**Figure 8:**
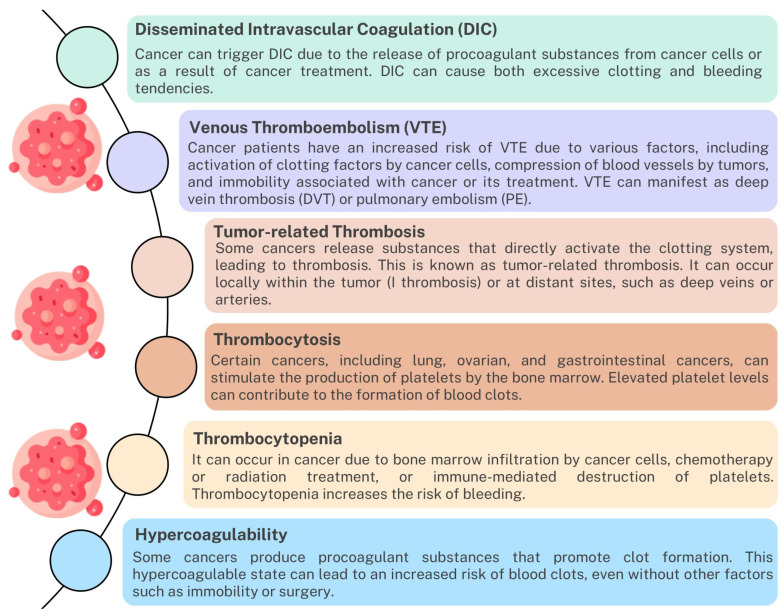
The impact of cancer on coagulation disorders based on [230,231,232].

The consequence of the events presented in Figure 8 is the occurrence of organ dysfunction. Excessive clot formation and consumption of coagulation factors and platelets in sepsis can impair blood flow to vital organs, leading to organ dysfunction [183,184]. Microcirculatory thrombosis and ischemia may further perpetuate the inflammatory response and contribute to multi-organ failure, which is life-threatening for patients [185]. From a clinical perspective, managing bleeding disorders in sepsis is challenging but critical to patient outcomes. Therefore, it is essential to monitor various biomarker molecules related to the coagulation system, such as D-dimer and prothrombin time (PT), elevated levels of which indicate coagulation disorders and can help guide treatment decisions [186,187,188,189]. Treatment strategies may include administering anticoagulants such as heparin to prevent further clot formation. However, the balance between preventing clot formation and avoiding bleeding complications must be carefully considered. In addition, treating the underlying infection, supporting organ function, and controlling the inflammatory response are essential components of sepsis treatment [190,191,192].

### 4.4. The Role of Coagulation Disorders in Autoimmune Diseases

The coagulation system plays a role in several disorders of the immune system, where abnormalities in coagulation may contribute to the pathogenesis of selected autoimmune diseases. In autoimmune diseases, an abnormal immune response can attack components of the coagulation system, leading to coagulation disorders and an increased risk of thrombosis. Recognition and treatment of these coagulation disorders are important to optimize patient care and reduce the risk of complications [193,194,195,196]. Table 3 presents selected immune system disorders and autoimmune diseases and their interactions with the coagulation system.

### 4.5. Disturbances of the Coagulation System in Neoplastic Diseases

The immune and coagulation systems have complex interactions in the context of cancer [224,225]. While the immune system plays a crucial role in identifying and eliminating cancer cells, it can also affect the coagulation system and vice versa. Studies indicate that the development and progression of cancer significantly affect the occurrence of coagulation disorders such as DIC, venous thromboembolism, thrombosis, thrombocytosis, thrombocytopenia, and hypercoagulability (Figure 8). Disturbances of this dynamic balance between the immune system and the coagulation system may contribute to the development of inflammation, angiogenesis, or excessive coagulation mediated by immune cells, which may affect the treatment process of patients [226,227,228,229].

#### 4.5.1. Development of Inflammation

Inflammation plays a key role in cancer, and its interaction with the coagulation system has been extensively studied. Inflammatory processes can promote tumor growth, angiogenesis (creating new blood vessels), and metastasis while affecting the coagulation system. During inflammation, immune cells release various pro-inflammatory molecules, including cytokines, chemokines, and growth factors. These factors can stimulate the production of TFs, a key initiator of the coagulation cascade. TF is expressed not only by immune cells but also by the tumor cells themselves, contributing to the procoagulant state. Furthermore, activated coagulation factors may exacerbate inflammation by recruiting immune cells to the site of injury or tumor, as observed in the tumor microenvironment characterized by immune cell infiltration [233,234,235,236,237]. An inflammatory response in the tumor microenvironment may also influence the immune response against the cancer. Inflammatory cells such as macrophages and neutrophils can secrete factors that create an immunosuppressive environment, impeding an effective antitumor immune response. In addition, inflammation-induced endothelial dysfunction may further contribute to coagulation disorders. Inflammatory mediators may disturb the balance between procoagulant and anticoagulant factors, leading to an increased tendency to form thrombi, which increases the risk of thromboembolic events. Cancer-associated thrombosis is thought to result from a complex interaction between inflammation, cancer cells, and the coagulation system [224,233,234,235,236,237].

#### 4.5.2. Angiogenesis

The formation of new blood vessels (angiogenesis) is crucial for tumor growth and metastasis. The immune and coagulation systems play a role in this process. Immune cells can release factors that promote angiogenesis, while the coagulation system contributes to the formation of blood vessels. Abnormal angiogenesis can lead to an imbalance in the coagulation system, resulting in excessive blood clot formation or bleeding [238,239,240,241].

#### 4.5.3. Coagulation Mediated by Immune Cells

One important aspect of immune cell-mediated coagulation in cancer is the activation of platelets and coagulation factors by immune cells such as macrophages and neutrophils that infiltrate the tumor microenvironment. These immune cells release pro-inflammatory molecules, including cytokines, chemokines, and TFs, which can trigger the coagulation cascade. Neutrophils release NETs (neutrophil extracellular traps), activating coagulation factors and promoting thrombosis. In addition to their role in hemostasis, platelets can interact with cancer cells and immune cells to influence the immune response and encourage coagulation [232,242,243]. Cancer cells can also express TF, a protein involved in initiating blood coagulation that can directly activate the coagulation pathway. Cancer cells’ expression of TFs is associated with an increased risk of thrombosis in cancer patients. The interaction between activated immune cells, platelets, and coagulation factors can lead to thrombosis, which the literature indicates is a significant cause of morbidity and mortality in cancer patients. In addition, the coagulation process can also influence tumor progression and metastasis. The formation of blood clots can provide a physical scaffold for the migration of cancer cells and facilitate their spread to distant organs. Coagulation factors such as fibrin may promote cancer cell survival and protect them from immune surveillance [68,244,245,246]. 

### 4.6. Immunotherapy and Coagulation

Immunotherapy is a promising approach to cancer treatment that uses the body’s immune system to recognize and attack cancer cells. Although immunotherapy has significantly benefited many patients, it can also affect the clotting process and potentially lead to some clotting-related effects [224,247]. The first is immune-related adverse events (irAEs) associated with immunotherapy, which may include thromboembolic events such as DVT or PE, reported in a small percentage of patients treated with inhibitor-based immunotherapy checkpoints [248,249,250]. The second process involves activating immune cells, such as T and natural killer cells, to target cancer cells. These activated immune cells can release pro-inflammatory cytokines and other factors indirectly affecting the coagulation system. Elevated levels of cytokines such as IL-6 are associated with an increased risk of thrombosis [243,251,252,253,254]. Immunotherapy can also lead to endothelial dysfunction, which refers to impaired function of the cells lining the blood vessels. Endothelial dysfunction can disrupt the delicate balance between the coagulation and anticoagulant processes, potentially promoting a procoagulant state. It can also lead to the release of pro-inflammatory molecules, further contributing to the activation of the coagulation system. Research indicates that some cancers may activate specific clotting pathways to support their growth and evade the immune system. Immunotherapy can interfere with these tumor-associated coagulation pathways, potentially affecting the balance of the coagulation system. This disruption can affect clotting and bleeding tendencies [255,256,257].

The exact mechanisms behind these events are not fully understood but may include immune dysregulation and the interplay between the immune and coagulation systems. The mechanisms underlying this relationship are still being investigated. Understanding the intricate relationship between the immune and coagulation systems in cancer is critical to developing effective treatments and managing complications. Healthcare professionals need to monitor both immune and coagulation parameters in cancer patients to ensure prompt intervention and appropriate management of any immune or coagulation abnormalities that may occur during the course of the disease or its treatment.

## 5. Conclusions

In conclusion, the intricate relationship between the coagulation and immune systems holds important clinical and research implications. Understanding the mutual interactions and crosstalk between these systems is crucial for comprehending the pathophysiology of various diseases and developing effective therapeutic strategies.

The immune system not only plays a key role in pathogen recognition and defense but also actively participates in the regulation of coagulation. Immune cells, including macrophages, neutrophils, and dendritic cells, contribute to coagulation by producing pro-inflammatory cytokines and expressing TFs. Additionally, certain immune cells, such as mast cells and basophils, release heparin, an anticoagulant that helps regulate coagulation. Conversely, the coagulation system actively interacts with the immune system, influencing immune responses. Coagulation factors, such as thrombin, have the ability to activate immune cells and enhance the production of pro-inflammatory cytokines. Fibrin, the primary component of blood clots, acts as a scaffold for immune cells, facilitating their recruitment and activation at sites of injury or infection. Coagulation factors can also directly impact the function of immune cells, including T cells and dendritic cells, affecting their migration, proliferation, and antigen presentation. Recognizing the intricate interplay between coagulation and the immune system has important implications for clinical practice and research. It emphasizes the need for a multidisciplinary approach in the management of clotting and immune-related conditions, such as sepsis, autoimmune diseases, and cancer. Furthermore, targeting the shared regulatory mechanisms between these systems holds promise for the development of novel therapeutic strategies to tackle these complex disorders. In terms of therapeutics, anticoagulation or antiplatelet drugs have demonstrated significant benefits. Anticoagulants, such as direct oral anticoagulants or warfarin, are effective in preventing thrombotic events by inhibiting specific clotting factors. By reducing the risk of thrombosis, these drugs can potentially prevent serious complications such as stroke, deep vein thrombosis, or pulmonary embolism. Antiplatelet drugs, such as aspirin or P2Y12 inhibitors, have proven beneficial in the prevention of cardiovascular events. By inhibiting platelet activation and aggregation, these drugs can reduce the risk of myocardial infarction, stroke, or peripheral arterial disease. However, it is essential to consider the delicate balance between coagulation and immune responses when utilizing these medications. Anticoagulant therapy carries inherent risks of bleeding complications, and antiplatelet drugs may impact immune responses and inflammation. Therefore, a careful assessment of individual patient factors and a personalized approach to therapy are crucial to optimize the benefits of these medications while minimizing potential risks. By drawing conclusions regarding the risks and potential benefits of anticoagulation or antiplatelet drugs, we underscore the importance of careful consideration in clinical decision-making. This comprehensive understanding of the interplay between coagulation and the immune system provides a foundation for tailored therapeutic strategies that maximize efficacy while minimizing adverse effects.

## Figures and Tables

**Table 1:** Characterization of platelet receptors involved in maintaining normal hemostasis functions.

Receptor Name	Gene	Protein ID	Molecular Weight [kDa]	Amino Acid Length	Ligands	Ref.
Platelet glycoprotein IX	*GP9*	P14770	19.046	177	✓ vWF, ✓ Thrombospondin-1, ✓ Thrombin, ✓ Factors XI, XII, ✓ P-selectin	[50]
Platelet glycoprotein Ib alpha chain	*GP1BA*	P07359	71.540	652		[51]
Platelet glycoprotein V	*GP5*	P40197	60.959	560		[52]
Platelet glycoprotein VI	*GP6*	Q9HCN6	36.866	339	✓ Collagen, ✓ Laminina	[53]
Integrin alpha-2/beta-1	*ITGA2*	P17301	129.296	1181	✓ Collagen, ✓ Laminina	[54]
Integrin alpha-6/beta-1	*ITGA6*	P23229	126.606	1130	✓ Laminina	[55]
Integrin alpha-V/	*ITGAV*	P06756	116.038	1048	✓ Vitronectin, ✓ Cytotoxin, ✓ Fibronectin, ✓ Fibrinogen, ✓ Laminin, ✓ Matrix metalloproteinase-2, ✓ Osteopontin, ✓ Osteomodulin, ✓ Prothrombin, ✓ Thrombospondin, ✓ vWF	[56]
Integrin alpha-IIb/beta-3	*ITGA2B*	P08514	113.377	1039	✓ Fibronectin, ✓ Fibrinogen, ✓ Plasminogen, ✓ Prothrombin, ✓ Thrombospondin, ✓ Vitronectin	[57]
Proteinase-activated receptor 1 (PAR1)	*F2R*	P25116	47.441	425	✓ Trombina	[58]
Proteinase-activated receptor 4 (PAR4)	*F2RL3*	Q96RI0	41.133	385		[59]
P2Y purinoceptor 1	*P2RY1*	P47900	42.072	373	✓ Extracellular adenine nucleotides such as ADP	[60]
P2Y purinoceptor 12	*P2RY12*	Q9H244	39.439	342	✓ ADP and ATP coupled to G-proteins	[61]
5-hydroxytryptamine receptor 2A	*HTR2A*	P28223	52.603	471	✓ 5-hydroxytryptamine (serotonin)	[62]

**Table 2:** Dysregulation of monocyte functioning in the course of selected thrombotic diseases.

Disease	Description of the Disorder	Ref.
Deep Vein Thrombosis (DVT) and Pulmonary Embolism (PE)	✓ DVT occurs when blood clots form in the deep veins, usually in the legs, while PE occurs when these clots travel and travel to the lungs. ✓ Under these conditions, monocytes may contribute to thrombus formation and propagation through increased TF expression and release of pro-inflammatory cytokines. ✓ Dysregulated monocyte function can lead to an imbalance in the coagulation system, promoting thrombus formation and the risk of embolism.	[74,89,90,91,99]
Arterial Thrombosis	✓ Arterial thrombosis can occur in conditions such as atherosclerosis, myocardial infarction, and stroke. ✓ Monocytes play a role in the pathogenesis of atherosclerosis by infiltrating the arterial wall, contributing to inflammation, and promoting atherosclerotic plaque formation. ✓ Under these conditions, dysregulated monocyte function can increase atherosclerotic plaque instability and thrombus formation, potentially triggering acute cardiovascular events.	[92,93]
Disseminated Intravascular Coagulation (DIC)	✓ DIC is a condition characterized by extensive coagulation system activation, leading to the formation of small blood clots throughout the body. ✓ In DIC, dysregulated monocyte activation and increased release of TF and pro-inflammatory cytokines may contribute to the overactivation of the coagulation cascade. ✓ This can result in wear of coagulation factors, platelets, and other blood components, leading to both excessive clotting and an increased bleeding tendency.	[94]
Antiphospholipid Syndrome (APS)	✓ APS is an autoimmune disease characterized by the presence of antiphospholipid antibodies. ✓ These antibodies can bind to the surface of cells, including monocytes, and interfere with their normal function. ✓ Dysfunctional monocytes in APS may contribute to an imbalance in the coagulation system, leading to an increased risk of arterial and venous thrombosis.	[95,96]
Thrombotic Microangiopathies	✓ Thrombotic microangiopathies are a group of disorders characterized by the formation of small blood clots in the microcirculation system, leading to organ damage. ✓ Dysregulated monocyte activation and increased release of pro-inflammatory mediators may contribute to the endothelial damage and platelet activation seen in these conditions, further promoting microvascular thrombus formation.	[97,98]

**Table 3:** Selected disorders of the immune system and their interactions with the coagulation system.

Disease	Description of the Disorder	Ref.
Systemic lupus erythematosus (SLE)	✓ SLE is an autoimmune disease characterized by the production of autoantibodies directed against various body components, including DNA, proteins, and phospholipids. ✓ In SLE, antiphospholipid antibodies (aPL) can lead to an increased risk of thrombosis, known as antiphospholipid syndrome (APS). ✓ The binding of aPL to phospholipids impairs the normal function of the coagulation system and endothelial cells, leading to a prothrombotic state and an increased risk of clot formation.	[197,198,199,200,201,202]
Rheumatoid arthritis (RA)	✓ RA is an autoimmune disease characterized by chronic inflammation, mainly affecting the joints. ✓ Inflammation in RA can activate the coagulation system, leading to an increased risk of thrombosis. ✓ Furthermore, patients with RA have an increased risk of developing cardiovascular disease, and coagulation dysregulation is responsible for the increased cardiovascular risk.	[203,204,205,206]
Antiphospholipid syndrome (APS)	✓ APS is an autoimmune disease characterized by antiphospholipid antibodies (aPL) that target phospholipid-binding proteins. ✓ These antibodies interfere with the normal functioning of the coagulation system and endothelial cells, leading to an increased risk of thrombosis. ✓ APS can cause arterial and venous blood clots and is associated with recurrent pregnancy losses and complications.	[207,208,209]
Systemic sclerosis (SSc)	✓ SSc, or scleroderma, is an autoimmune disease characterized by fibrosis and vascular abnormalities. ✓ The vascular abnormalities seen in SSc may lead to endothelial damage and dysfunction, predisposing patients to thrombotic events. ✓ In addition, autoantibodies directed against phospholipids, such as antibodies against cardiolipin, may be present in SSc, potentially contributing to coagulation disorders.	[210,211,212,213,214]
Inflammatory bowel disease (IBD)	✓ IBD, which includes Crohn’s disease and ulcerative colitis, is characterized by chronic digestive tract inflammation. ✓ Inflammation in IBD can trigger coagulation system activation, leading to an increased risk of thromboembolic events. ✓ Pro-inflammatory cytokines released during IBD may promote endothelial dysfunction and prothrombotic changes, contributing to the thrombotic complications seen in some IBD patients.	[215,216,217]
Immune thrombocytopenic purpura (ITP)	✓ ITP is an autoimmune disorder characterized by a low platelet count (thrombocytopenia) due to the production of autoantibodies directed against platelets. ✓ The immune-mediated destruction of platelets in ITP can lead to coagulation disorders. ✓ However, in some cases, paradoxical activation of the coagulation system may occur in response to the destruction of platelets, leading to small blood clots.	[218,219,220]
Limfohistiocytoza hemofagocytarna (HLH)	✓ HLH is a life-threatening hyperinflammatory syndrome characterized by uncontrolled immune activation. ✓ In HLH, an overactive immune response can trigger the release of pro-inflammatory cytokines such as IFN-γ, TNF-α, and IL-6, which can promote endothelial activation and induce a prothrombotic state.	[221,222,223]
